# The burden and types of anaemia among HIV infected, ART-naive injection substance users in Kenya

**DOI:** 10.4314/ahs.v22i1.52

**Published:** 2022-03

**Authors:** Emmanuel Mulaya Khazalwa, Tom Were, David Hughes Mulama, Valentine Budambula

**Affiliations:** 1 Department of Biological Sciences, Masinde Muliro University of Science and Technology, Kakamega, Kenya; 2 Department of Medical Laboratory Science, Masinde Muliro University of Science and Technology, Kakamega, Kenya; 3 Department of Environment and Health Sciences, Technical University of Mombasa, Mombasa, Kenya

**Keywords:** Anaemia, HIV/AIDS, substance use

## Abstract

**Introduction:**

Illicit substance use and HIV infection cause haematological derangements. Anaemia characterized by a reduction in the quality and quantity erythrocytes is the most common disorder in both HIV-positive persons and illicit substance users.

**Objective:**

To describe anaemia burden, types, and its association with HIV in injectable substance users in Mombasa, Kenya.

**Methods:**

This descriptive case-control study evaluated red cell indices and morphology in 494 adults. The primary outcome was anaemia. The association of anaemia with HIV in injection substance users was determined using the chi-square test.

**Results:**

The participants included 275 injection substance users (ISU), (HIV-positive, n=62 and HIV-negative, n=213); and 219 non-injection substance users (nonISU), (HIV-positive, n=33 and HIV-negative, n=186). Overall, 49% were anaemic with anaemia burden significantly differing across the groups, X2(3, N=494) =12.1, p=0.0070. Anaemia burden was higher in HIV-positive ISU compared to HIV-negative ISU (odds ratio (OR) = 1.59, 95% confidence interval (CI): 0.85, 2.96); and HIV-positive nonISU compared to HIV-negative nonISU (OR = 0.37, 95% confidence interval (CI): 0.17, 0.79). Most of the anaemia was dimorphic in both HIV-positive (ISU, 67% and nonISU, 52%) and HIV-negative (ISU, 43% and nonISU, 55%) participants.

**Conclusion:**

Infection with HIV is associated with increased risk of anaemia in injectable and non-injectable substance users. Majority of the anaemia was dimorphic suggestive of multiple aetiologies. Establishing the related aetiologies is essential for the effective treatment of anaemia. The accurate evaluation of thin blood films remains an essential tool in diagnosing an array of haematologic disorders and as a reference for further tests and patient management.

## Introduction

The use of illicit substances and the non-medical use of prescription medicines is an increasing global health problem^1^. Reports by the United Nations Office on Drugs and Crime (UNODC) indicate that 5.6% of the world's population aged 15–64 years had an illicit substance debut in 2016^2^,^3^. Illicit substance use causes several ‘substance use disorders’ which are associated with life-threatening medical conditions such as anaemia, substance dependence and blood-borne infections such as the human immunodeficiency virus (HIV), the hepatitis B virus (HBV) and the hepatitis C virus (HCV)^4^, ^29^,^30^ In 2018, the UNODC reported that 450,000 deaths in 2015 were directly attributable to substance use^5^. Illicit substance use is also implicated in the soaring HIV burden and HIV disease progression^6^. Statistics from the UNODC in 2018 showed that 12.5% of illicit injection substance users were positive with HIV by the end of 2017^7^. In addition, HIV transmission rates are higher in illicit substance users compared to the general population^8^. Furthermore, people who inject drugs (PWID) constitute 30% of new HIV infections in the world^9^,^10^. The underlying factors influencing the HIV burden include sharing hypodermic needles, flashing blood and high-risk sexual practices amongst substance users^11^.

Illicit substance use and HIV infection are implicated in hematologic derangements^12^. Anaemia is the most frequent hematologic manifestation in both the HIV positive^13^ and illicit substance users^14^. Whereas studies implicate anaemia as the prevalent hematologic disorder, little is known about the burden and types of anaemia in injectable substance users. Anaemia in HIV-positive persons is life threatening as it is associated with enhanced HIV disease progression and increased mortality and morbidity^28^. Substance use is also associated with haematological derangements including anaemia. Some of the interventions undertaken to manage and treat anaemia have yielded little or no results even after prolonged management/treatment^31^,^32^. This necessitated the need to understand the types of anaemia for effective management and treatment. The World Health Organization (WHO) laboratory guidelines recommend the establishment of haemoglobin concentration and the evaluation of use of complete blood count (CBC) in diagnosing anaemia^15^. This method is prone to bias as the blood parameters are altered with variations in plasma volume without changes in the erythrocyte mass^16^, as well as the body's ability to compensate for blood loss during the onset of anaemia^17^. Moreover, CBC does not amply reveal the morphological changes in blood cells, which are important in elucidating primary and secondary blood disorders and their pathophysiology. To overcome these drawbacks, we combined the CBC and the microscopic examination of thin blood films to adequately diagnose and characterize anaemia in HIV infected injection and non-injection substance users.

## Materials and methods

### Study design and study site

This descriptive clinical case-control study was conducted as part of a wider study investigating the sociodemographic, nutritional and microbiologic determinants of HIV infections among injection substance users in Mombasa County^11^. Further information about the study area has been previously published^18^.

### Study population

The study population comprised of 494 adults (males, n=329 and females, n=165) aged between 18 and 65 years. The study subjects were grouped as follows: 1) HIV-positive injection substance users (HIV+ISU, n=62); 2) HIV-negative injection substance users (HIVISU, n=213); 3) HIV-positive non-injection substance users (HIV + nonISU, n=33) and 4) HIV-negative non-injection substance users (HIV-nonISU). Injection substance users were individuals having visible needle scars and having reported using an injection substance at least once in the previous month. Participants were classified as either HIV-seropositive (cases) or HIV-seronegative (controls) using the DetermineTM HIV test kit. All the HIV-positive participants recruited into this study had not been previously initiated to antiretroviral therapy. A detailed description of the sampling techniques and study population have been published^11^.

### Ethical considerations

Ethical approvals for the study were obtained from the Kenyatta University (Protocol KU/R/COMM/51/32-4) and the Masinde Muliro University of Science and Technology (Protocol MMU/COR: 403012-vol2[8]) institutional review board (IRB). All the respondents were exhaustively educated as per the internationally recommended guidelines^19^ and written informed consent obtained prior to enrolment.

### Collection of blood samples

Venous blood samples were collected from the freely consenting participants by a certified phlebotomist using a Vacutainer assembly into 5ml EDTA vacutainerTM tubes (BD, Franklin Lakes, USA). Blood was collected between 8.00am and 10.00am to obtain strictly comparable values and control for diurnal variations in blood parameters^20^. All laboratory tests were performed within the hour of sample collection to maintain sample integrity.

### Experimental procedures Erythrocyte indices

Complete Blood Counts (CBC) were obtained using the quantitative BC-3200 Mindray auto-haematology analyser (MindrayTM Inc., Mahwah, USA) as outlined in the Centres for Disease Control guidelines^21^. The erythrocyte indices: 1) red blood cell count (RBC, ×1012/L); 2) haemoglobin concentration (Hb, g/dL); 3) haematocrit (HCT, %); 4) mean corpuscular volume (MCV, fL); 5) mean corpuscular haemoglobin (MCH, pg.); 6) mean corpuscular haemoglobin concentration (MCHC, g/dL); and 7) the red cell distribution width (RDW, %) were recorded. The RBC, HB and HCT were used to determine the severity of anaemia. MCV, MCH, MCHC, RDW together with findings from the examination of peripheral blood films for red cell morphology were used to type the anaemia.

### Preparation and examination of thin blood film (BF) for red cell morphology

Duplicate thin blood films were made on new microscope slides (Thermo-ScientificTM) to prevent cell aggregation and stain precipitation. The prepared films were Leishman stained^22^. Briefly, the freshly prepared blood films were thoroughly air-dried and fixed in absolute methanol for 2 minutes. Afterwards, the blood films were flooded with undiluted Leishman stain for a few seconds. The stain was then diluted with buffered water (pH. 6.8) and left to stand for 10 minutes for differentiation. The stained blood films were then washed off under a gentle stream of running tap water to remove excess stain. The back of the slide was wiped, and the blood films placed standing on a draining rack to dry. Once dried, two independent blinded hematologists assessed the blood films for erythrocyte morphology and recorded their findings on a standard RBC morphology form.

Slides with extreme variations in the results of the two haematologists were re-read by a third independent haematologist. Ten per cent (n=50) of the read slides were randomly selected and the results confirmed by a pathologist.

### Assessing anaemia Anaemia burden

Anaemia was defined as haemoglobin (Hb) concentration less than 13.0g/dL and 12.0g/dL in males and females, respectively^15^. The burden of anaemia was defined as the ratio of the occurrences of anaemia to the number of sampled individuals within each study group.

### Anaemia severity

Anaemia severity was classified based on haemoglobin concentration according to the world health organiza tion (WHO) guidelines: mild anaemia (Hb11.0-12.9g/dL and Hb 110-11.9g/dL in males and females respectively), moderate anaemia (Hb 8.0-10.9g/dL) and severe anaemia (Hb<8.0g/dL)^15^.

### Anaemia types

The cytometric classification of anaemia was conducted using erythrocyte indices (MCV, MCHC and MCH) and erythrocyte morphology (e.g., pigmentation, pencil cells, poikilocytosis, spherocytosis etc). For example, macrocytic anaemia, (MCV>100fl); microcytic anaemia, (MCV<80fl); normocytic anaemia, (MCV 80fl-100fl); hyperchromic anaemia, (MCHC>36g/dL); hypochromic anaemia, (MCHC<31g/dL) and normo chromic anaemia, (MCHC 31g/dL-36g/dL)^23^. These classifications were confirmed to be consistent with Leishman-stained blood film readings. Erythrocyte morphologies associated with anaemia were categorized as follows:

**Dimorphic anaemia:** Prescence of dimorphic erythrocytes i.e., observation of an admixture of erythrocyte populations in the blood smear (e.g. hypochromic microcytes with either normochromic normocytes or hypochromic/normochromic macrocytes)^24^ with double peaks in the red blood cell distribution graph^25^ as associated with combined iron deficiency and nutritional macrocytic anaemia.

Anaemia of inflammation: Prescence of rouleaux, tear-drop cells and poikilocytes in the blood smear and normal RDW (11.6–13.4%) with either 1) normal (80–100fl) or reduced (<80fl) MCV; 2) normal (25–33pg) or reduced MCH (<25pg); and 3) normal (31–36g/dL) or reduced MCHC (<31g/dL); as presented in patients with anaemia of inflammation^26^,^27^.

Anaemia of vitamin deficiency: Prescence of hypo-pigmented microcytes with narrow-elongated erythrocytes (pencil cells, reduced MCHC (<31g/dl), reduced MCV (<80fl), elevated RDW (>13.4%) as observed in iron deficiency anaemia^28^.

Secondly, presence of oval-macrocytes, megaloblasts, poikilocytosis and neutrophil hyper-segmentation in blood smears with elevated MCV (>110fl) and RDW (>13.4%) in CBC profiles as observed in patients with confirmed vitamin B12 / folate deficiency anaemia^29^,^30^.

**Haemolytic anaemia:** Prescence of schistocytes, basophilic stippling, nucleated erythrocytes, spherocytosis, reticulocytosis, polychromasia with half-ghost cells and erythrocytes with “bitten out” margins, and sickle cells in the blood smear^31^. Additionally, we assessed the full blood count for reduced MCV (<80fl), normal MCV (80–100fl), raised MCH (>33pg) and MCHC (>36g/dL) as seen in haemolytic anaemia^32^. Extensive spherocytosis with reduced MCV and elevated MCH was used as a marker of autoimmune haemolytic anaemia while extensive schistocytosis with normal MCV was used as a marker of microangiopathic haemolytic anaemia.

### Body mass index (BMI)

Anthropometric measures were obtained from each study participants at enrolment as per the Centres of Disease Control guidelines^33^. Height (m) was measured to the nearest 0.1 cm using the Health-o-meter PORTROD wall mounted height rod (Health O meter ®, McCook, USA). Study participants were weighed in kilograms (kg) using a portable digital weight scale (Rich forth Electronics Co., Fuzhou, China). The BMI was calculated using the height and weight measurements of the study participants using the formula:

$$\mathrm{BMI}\;(\mathrm{Kg}/m^{2})=\frac{\mathrm{Weight}(\mathrm{Kg})}{\mathrm{Height}(m)}$$

BMI < 18.5 was classified as underweight.

### Statistical analysis

Statistical analyses were conducted in GraphPad Prism Version 6.01 (©2012GraphPad Software, Inc.). We used Chi-square test to determine the association between anaemia and HIV infection in injectable and non-injectable substance users. The continuous variables such as weight and height were compared across the groups using a one-way ANOVA test. Erythrocyte measures were compared using non-parametric ANOVA (Kruskal-Wallis Test) followed by Dunn's post-hoc corrections for multiple comparisons to control for the overall type-I error. All tests were two-tailed and p values <0.05 was considered statistically significant.

## Results

### Baseline characteristics

The baseline characteristics of the study participants are presented in Table 1. A total of 494 adults (males, n=329 and females, n=165) were recruited into the study. The number of participants that were substance users significantly differed by gender, X2 (3, N = 494)=120.4, p <0.0001. One-way analysis of variance showed that the effect of age was not significant across the study groups F (3, 490) =2.551, p=0.055.

**Table 1:** Age, height, and weight of the study participants

	Non-Injection Substance Users		Injection Substance Users		
			
Characteristics	HIV-nonISU, n=186	HIV+nonISU, n=33	HIV-ISU, n=213	HIV+ISU, n=62	p-Value
**Gender, n (%)**					
Females	103 (55)	17 (52)	14 (7)	31 (50)	**<0.0001**
Males	83 (45)	16 (49)	199 (93)	31 (50)	
**Demographic, M(IQR)**					
Age, yrs.'	31.2 (11.9)	34.2 (14.7)	31.7 (9.1)	30.6 (6.3)	0.055
Height (meters)	1.67(0.11)	1.67 (0.13)	1.71 (0.09)	1.69 (0.14)	**<0.0001**
Weight (kg)	61 (12.3)	52 (14.0)	54 (9.0)	54 (8.5)	**<0.0001**
**BMI<18.5, n (%)**	42 (23)	16 (49)	101 (47)	20 (32)	
**BMI≥18.5, n (%)**	144 (77)	17 (51)	112 (53)	42 (68)	
**Duration of use**					
≥1 year, n (%)	41 (22)	7 (21)	143 (67)	45 (73)	
<1 year, n (%)	7 (4)	3 (9)	70 (33)	17 (27)	
**Frequency of use**					
>1 daily, n (%)	48 (26)	7 (21)	201 (94)	49 (79.0)	
≤1 daily, n (%)	12 (7)	3 (9)	12 (6)	13 (21.0)	

Data are presented as number (n) and proportion (%) of subjects for gender, BMI, duration, and frequency of substance use. Age, height, and weight are presented as median (interquartile range, IQR). IQR=the difference between the 75^th^ and 25^th^ quartile. Gender was compared across the study groups using the Chi-square test while demographic data analysed using one-way ANOVA. Significant p-values are in bold. HIV+ISU+, HIV-positive injection substance users; HIV-ISU+, HIV-negative, injection substance users; HIV+ISU-, HIV-positive non-injection substance users; HIV-ISU-, HIV-negative non-injection substance users.

Participant's height (m) and differed across the study groups F (3, 490) =10.22, p<0.0001). Similarly, weight (kg) also differed across the groups F (3,490) = 9.39, p<0.0001 being higher in HIV-ISU compared to HIV+nonISU (p<0.0025), HIV-ISU (p<0.0001) and HIV+ISU (p<0.0001). Evaluation of the BMI showed that 32% of the HIV+ISU were underweight compared to 47% of the HIV-ISU X2 (1, N = 275) =4.5, p =0.0343. However, 49% of the HIV+nonISU were underweight compared to 23% of the HIV-nonISU X2 (1, N = 219) =9.7, p <0.0019.

Approximately 73% of HIV+ISU reported to have used injectable substances for more than a year compared to 67% HIV-ISU X2 (1, N = 275) =0.7, p=0.4172. Amongst the non-injectable substance users, 21% HIV + non ISU and 22% HIV-non ISU reported to have used substances for more than year X2 (1, N = 58)=1.4, p =0.2404.

Assessment of the frequency of substance use showed that 79% of HIV+ISU reported to use the substances more than once daily compared to 94% of HIV-ISU X2 (1, N = 275) =13.7, p =0.0002. In contrast, 21% of HIV+nonISU and 26% of HIV-nonISU reported to have used the substances more than one daily X2 (1, N = 70) =0.5, p =0.4755.

### Erythrocyte measures in substance users

Erythrocyte measurements are summarized in Table 2. The median RBC counts significantly differed across the groups H (3,490) =4.9, p=0.0029. Post hoc analyses using the Dunn's multiple comparisons test showed that the medial RBC count was higher amongst the HIV-ISU compared to the HIV+ISU (p=0.4062). Similarly, HIV-nonISU exhibited elevated RBC counts compared to the HIV+nonISU (p=0.1793). Significantly high RBC counts was observed amongst HIVISU compared to HIV+nonISU (p=0.0047).

**Table 2:** Erythrocyte indices and anaemia

	Non-Injection Substance Users		Injection Substance Users		
			
Characteristics	HIV-nonISU, n=186	HIV+ nonISU, n=33	HIV-ISU, n=213	HIV+ISU, n=62	p-value
**Indices, median (IQR)**					
RBC, ×10^12^/L	4.8 (0.9)	4.0 (1.2)	4.9 (0.8)	4.7 (0.7)	**0.0029**
Hb, g/dL	12.8 (2.5)	11.6 (3.3)	12.6 (2.3)	12.5 (1.8)	**<0.0002**
MCV, fL	84.9 (10.7)	86.1 (17.3)	85.2 (8.8)	85.9 (9.5)	0.0726
MCH, pg.	27.3 (4.4)	27.6 (6.2)	26.4 (3.7)	27.0 (4.4)	0.1810
MCHC, g/dL	31.9 (2.7)	31.5 (4.2)	30.7 (3.0)	31.3 (2.7)	**<0.0001**
RDW, %	14.7 (3.2)	13.9 (2.9)	13.5 (2.3)	14.0 (3.0)	**0.0036**
**Anemia, n (%)**	73 (39)	21 (64)	120 (56)	30 (48)	**0.0023**
Mild	46 (63)	7 (33)	80 (67)	17 (57)	-
Moderate	21 (29)	11 (52)	39 (33)	12 (40)	-
Severe	6 (8)	3 (14)	1 (1)	1 (3)	-

Data are presented as median (interquartile range, IQR) for erythrocyte indices; and quantity (n) and proportion (%) for anaemia. **HIV+ISU**, HIV positive injection substance users; **HIV-ISU**, HIV negative, injection substance users; **HIV+nonISU**, HIV positive non-injection substance users; **HIV-nonISU**, HIV negative non-injection substance users; **RBC**, red blood cell; **HB**, Haemoglobin; **MCV**, mean corpuscular volume; **MCH**, mean corpuscular haemoglobin; **MCHC**, mean corpuscular haemoglobin concentration; RDW, red cell distribution width. **Mild anaemia** (Hb11.0-12.9g/dL and Hb 110-11.9g/dL in males and females respectively); **Moderate anaemia** (Hb 8.0-10.9g/dL) and **Severe anaemia** (Hb<8.0g/dL) (WHO, 2011). IQR=the difference between the 75^th^ quartile and the 25^th^ quartile. Indices were compared using Kruskal-Wallis Test followed by Dunn's post-hoc corrections for multiple comparisons. Anaemia was assessed using the Chi-square test. All tests were two-tailed and p values <0.05 considered statistically significant.

The medial haemoglobin concentrations were differing across the study groups H (3,490) =8.6, p=0.0002. Dunn's multiple comparisons test showed that haemoglobin concentration was higher in HIV-nonISU compared to HIV+nonISU (p=0.0001). Additionally, HIV+nonISU exhibited significantly lower haemoglobin concentration compared to the HIV-ISU (p=0.0035). However, no significant changes in the medial haemoglobin concentration were observed amongst the HIV-ISU and HIV+ISU (p>0.9999).

The MCV and MCH did not significantly vary across the study groups. The MCHC however, differed across the study groups (p<0.0001) being lower in HIV-ISU comparative to HIV-nonISU (p<0.0001). The RDW varied across the study groups (p=0.0024) with increased anisocytosis observed in HIV-nonISU compared to HIV-ISU (p=0.0020).

### Anaemia burden and severity

**Figure 1:**
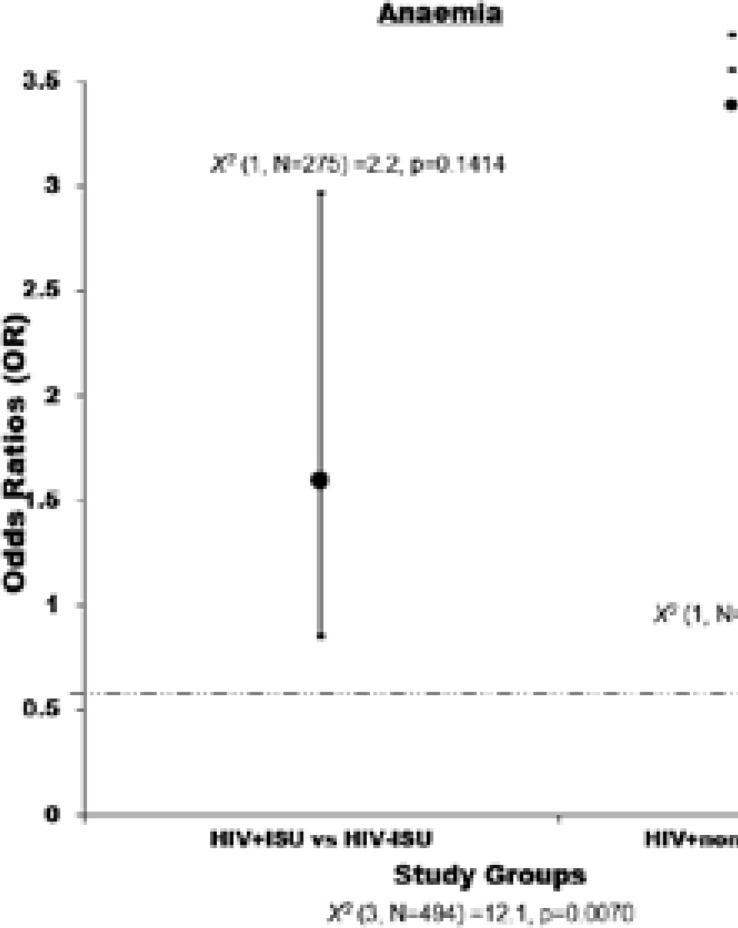
Chi-square test for the association between anaemia and HIV in substance users. **HIV+ISU**, HIV positive injection substance users; **HIV-ISU**, HIV negative, injection substance users; **HIV+nonISU**, HIV positive non-injection substance users; **HIV-nonISU**, HIV negative non-injection substance users.

The burden and severity of anaemia are presented in Table 2. Overall, 49% of the participants were anaemic with anaemia burden significantly differing across the groups, X2 (3, N=494) =12.1, p=0.0070. Anaemia burden was higher in HIV+nonISU compared to HIV-nonISU (odds ratio (OR) = 0.37, 95% confidence interval (CI): 0.17–0.79, p=0.0091). Likewise, the burden of anaemia was higher amongst HIV+ISU compared to HIV-ISU (OR = 1.59, 95% CI: 0.85–2.97, p=0.1742) (Figure 1). Most of the anaemia was mild (60%) and moderate (35%) (HIV+ISU, 56.7% and 40%, and HIV-nonISU, 33% and 52.4%; HIV-ISU, 66.7% and 32.5%, and HIV-nonISU, 63% and 28.8%), respectively.

### Anaemia types

The most prevalent types of anaemia were hypochromic (54.6%) and normochromic (43.8%) anaemia: (HIV-positive ISU, 50% and 46.7%; and nonISU, 61.9% and 38.1%; HIV-negative ISU, 63.3% and 35%, and nonISU 39.7% and 60.3%). Hyperchromic anaemia (1.5%) was less common manifesting amongst the HIV+ISU (3.3%) and the HIV-ISU (1.7%). Morphologically, normocytes (66.5%) were the most prevalent followed by microcytes (31.5%) and macrocytes (1.9%). This is summarized in Table 3.

**Table 3:** Erythrocyte morphology of the anaemic individuals

	Non-Injection Substance Users		Injection Substance Users		
			
Morphology, n (%)	HIV-nonISU, n=73	HIV+ nonISU, n=21	HIV-ISU, n=120	HIV+ISU, n=30	Total, n=244
**Erythrocyte size**					
Normocytes	41 (56.2)	11 (52.4)	91 (75.8)	19 (63.3)	173 (66.5)
Microcytes	32 (43.8)	8 (38.1)	28 (23.3)	9 (30.0)	82 (31.5)
Macrocytes	0 (0.0)	2 (9.5)	1 (0.8)	2 (6.7)	5 (1.9)
**Hypochromic erythrocytes**	**29 (39.7)**	**13 (61.9)**	**76 (63.3)**	**15 (50.0)**	**142 (54.6)**
Normocytes	14 (19.2)	4 (19.0)	56 (46.7)	9 (30.0)	88 (62.0)
Microcytes	15 (20.5)	7 (33.3)	20 (16.7)	4 (13.3)	50 (35.2)
Macrocytes	0 (0.0)	2 (9.5)	0 (0.0)	2 (6.7)	4 (2.8)
**Normochromic erythrocytes**	**44 (60.3)**	**8 (38.1)**	**42 (35.0)**	**14 (46.7)**	**114 (43.8)**
Normocytes	27 (37.0)	7 (33.3)	33 (27.5)	9 (30.0)	81 (71.1)
Microcytes	17 (23.3)	1 (4.8)	8 (6.7)	5 (16.7)	32 (28.1)
Macrocytes	0 (0.0)	0 (0.0)	1 (0.8)	0 (0.0)	1 (0.8)
**Hyperchromic erythrocytes**	**0 (0.0)**	**0 (0.0)**	**2 (1.7)**	**1 (3.3)**	**4 (1.5)**
Normocytes	0 (0.0)	0 (0.0)	2 (1.7)	1 (3.3)	4 (100.0)

Data are presented as numbers (n) and proportions (%) for erythrocyte morphology. Hypochromic (MCHC<31g/dL); normochromic (MCHC 31g/dL-36g/dL); hyperchromic (MCHC>36g/dL); normocyte (MCV 80fl-100fl); microcyte (MCV<80fl); macrocyte (MCV>100fl); **HIV+ISU**, HIV-positive injection substance users; **HIV-ISU**, HIV-negative, injection substance users;

**HIV+nonISU**, HIV-positive non-injection substance users; **HIV-nonISU**, HIV-negative non-injection substance users.

The combined evaluation of the thin blood films and complete blood counts are as summarized in Table 4. The number of participants that were anaemic across the study groups did not differ by erythrocyte characterization, X2 (9, N=244) =13.23, p=0.1523.

**Table 4:** Cytometric classification of anaemia

	Non-Injection substance		Injection Substance		
			
Classification	HIV-nonISU n=73	HIV+nonISU n=21	HIV-ISU n=120	HIV+ISU n=30	Total n=244
**1). Dimorphic anaemia.**	**40 (55%)**	**11 (52%)**	**51 (43%)**	**20 (67%)**	**122 (50%)**
AD + HA	24 (60%)	8 (73%)	41 (80%)	14 (70%)	93 (72%)
AD+AI	14 (35%)	2 (18%)	9 (18%)	3 (15%)	29 (23%)
AD+MA	0 (0%)	1 (9%)	1 (2%)	3 (15%)	5 (4%)
HA+AI	2 (5%)	0 (0%)	0 (0%)	0 (0%)	2 (2%)
**2). Anaemia of inflammation.**	11 (15%)	3 (14%)	36 (30%)	5 (17%)	55 (23%)
**3). Anaemia due to vitamin** **deficiency.**	12 (16%)	5 (24%)	16 (13%)	4 (13%)	37 (15%)
**4). Haemolytic anaemia.**	10 (14%)	2			
(10%)	17 (14%)	1			
(3%)	30 (12%)				

Erythrocyte characteristics of anaemic participants. Proportions (%) have been calculated within each group. **AD-** anaemia of vitamin deficiency; **AI-** anaemia of inflammation; MB-megaloblastic anaemia; **HA-** haemolytic anaemia; **HIV+ISU**, HIV-positive injection substance users; **HIV-ISU**, HIV-negative, injection substance users; **HIV+nonISU**, HIV-positive non-injection substance users; **HIV-nonISU**, HIV-negative non-injection substance users

Dimorphic anaemia was the most prevalent across all the study groups. The most common type of dimorphic anaemia was characterized by hypochromic microcytes with normochromic normocytes (HIV-positive ISU, 90% and nonISU 82%; and HIV-negative ISU, 98% and nonISU, 100%). Hypochromic microcytes with normochromic macrocytes were observed in HIV-ISU (2%) and HIV+nonISU (98%). Hypochromic microcytes with hypochromic macrocytes were only observed in HIV+ISU (10%).

## Discussion

This study describes the burden and types of anaemia in injection and non-injection substance users from Mombasa Kenya. In Addition, a detailed characterization of erythrocyte morphology and the association of HIV with anaemia in injectable and non-injectable substance users are reported.

Our results show that the burden of anaemia was higher in the HIV-positive substance users compared to the HIV-negative substance users. HIV-positive injection and non-injection substance users were more likely to suffer anaemia compared to their HIV-negative counterparts. Moreover, anaemia severity was marked in injectable substance users compared to non-injectable substance users. Significantly low erythrocyte counts were observed in HIV-positive non-injection substance users compared to the HIV-negative non-injection substance users. Therefore, HIV infection coupled with substance use exacerbates anaemia. This is likely due to the synergistic effect of excessive oxidative stress on cells associated with substance use and the direct induction of apoptosis on haematopoietic cells by HIV. Also, HIV associated auto-immune reactions combined with the impeded iron absorption in the duodenum by some illicit substances such as alcohol and khat could be a factor. Other studies show that HIV anti-viral antibodies against the Gag fragment cross-react with erythropoietin 1 (EP1) resulting in impaired erythropoiesis and the consequent manifestation of anaemia^34^. A study in West Africa revealed that HIV is associated with abnormal erythrocyte quantities and qualities^35^. HIV has been shown to disturb the division and survival of hematopoietic progenitor cells^36^,^37^. Moreover, HIV infection of the bone marrow stromal cells has been documented to negatively influence the production of erythropoietin, a key hormone for erythropoiesis^34^,^38^. This is consistent with our laboratory findings where erythrocytes were significantly reduced amongst the HIV-positive participants. Likewise, haemoglobin levels were lower in HIV+nonISU compared to HIV-nonISU and the HIVISU. This outcome is coherent with a Ghanaian study that reported a decline in haemoglobin concentration as a marker of HIV-disease progression^39^, and folic acid deficiency which is concomitant with jejunal pathology in HIV-positive patients^38^.

Dimorphic anaemia was prevalent across all the groups indicative of multiple aetiologies of anaemia at different stages of progression. Most participants reported to have suffered from chronic recurrent and unresolved anaemia likely due to the manifold causes of anaemia. Erythrocyte morphology amongst these individuals were predominantly consistent with those seen in iron deficiency anaemia coupled with either 1) haemolytic anaema, 2) anaemia of inflammation and 3) vitamin B12/folate deficiency. A small proportion of this population had a combined erythrocyte morphology consistent with haemolytic anaemia and anaemia of inflammation. Furthermore, anaemia of vitamin/mineral deficiency was the third most common type of anaemia. Substance addicts avoid meals and fast to prolong the psychedelic effects of substances^40^. The participants in this study were from resource-limited backgrounds. They primarily spend whatever money they must to sustain their substance use habit. Consequently, they have poor intake of fruits, vegetables and animal products resulting in several vitamin deficiencies. Vitamins are necessary for haemoglobin synthesis (for example vitamin B12, folate) and the absorption of iron from the intestines (for example vitamin C)^41^,^42^. Moreover, nutritional deficiency could be because of substance-induced damage of the gastrointestinal mucosa. Mal-absorption states in these groups need to be investigated including the production and inhibition of the intrinsic factor, and examination bone marrow smears whch are important in differentiating real nutritional deficiencies from impaired nutrient absorption/utilization.

Anaemia of inflammation was the second most prevalent type of anaemia. Substance use has been shown to trigger the inflammatory responses. For example, khat and alcohol use are associated with intestinal lesions that promote gastritis^43^–^45^. This intestinal inflammation is likely to cause the liver to secrete more of the hormone hepcidin which inhibits the body from utilizing stored iron (ferritin) and reduces iron absorption in the duodenum^46^.

Haemolytic anaemia was the least frequent type of anaemia. Laboratory analyses showed that 13% of the participants possibly suffered either autoimmune haemolytic anaemia (observable spherocytosis) or microangiopathic haemolysis (observable schistocytosis). It is likely that the observed intravascular haemolysis was attributable to the damping effect where drug metabolites are adsorbed on erythrocytes which become antigenic resulting in their untimely splenic clearance by drug dependent/independent antibodies and macrophages^47^. Normochromic anaemia is also associated with the accelerated red blood cell turnover and suppression of red blood cell production even when there is adequate iron intake^48^. Further investigations are required to delineate heritable and acquired traits that predispose these groups to high erythrocyte turnover. For example, direct antiglobulin test (DAT), urine free haemoglobin test, urine hemosiderin test, erythrocyte survival test, cold agglutinin titre, glucose-6-phosphate dehydrogenase (G6PD) and sickle cell screen are required to differentiate heritable and acquired traits that predispose these groups to high erythrocyte turnover.

Over 50% of the anaemia was hypochromic. Normocytic-hypochromic and microcytic-hypochromic anaemia were the predominant hypochromic anaemias. A study by Dancheck et al.,^49^ demonstrated that normocytic hypochromic anaemia in HIV-positive and HIV-negative women was associated with the use of illicit injection substances resulting in iron deficiency^49^. We speculate that injection substance users suffer chronic inflammation due to frequent skin abscesses and vasculitis resulting in increased circulating inflammatory cytokines (e.g., Tumour necrosis factor alpha (TNFα)) that affect erythropoiesis and could contribute to microcytic hypochromic anaemia^50^,^51^. Normocytic normochromic anaemia is associated with chronic inflammation, erythrocyte annihilation and the desertion of erythrocyte precursors in the bone marrow^52^. HIV disease progression has been associated with the desertion of erythrocyte precursors in the bone marrow^53^,^54^. Substance use has been shown to activate the inflammatory response. For example, khat and alcohol use have been documented to cause intestinal lesions leading to gastritis^43^–^45^. This intestinal inflammation is likely to cause the liver to secrete more of the hormone hepcidin which inhibits the body from utilizing stored iron (ferritin) and reducing iron absorption in the duodenum^46^. It would be of interest to evaluate markers of inflammation such as the C-reactive protein (CRP) and procalcitonin (PCT) in these groups.

One potential limitation of this study is in its design. A longitudinal survey would be more informative in assessing the association and development of anaemia with other factors such as the duration, frequency and type of substance used. However, this limitation does not significantly impact our findings as the outcome would possibly be similar. Second, invitro cultures for erythropoiesis with physiological concentrations of the substances used would be informative in evaluating the specific erythropoietic pathways affected. Third, conducting reticulocyte counts would have given a more detailed evaluation of anaemia in the study population. However, we opted to analyse RBC histograms which are faster and as informative as the manual reticulocyte count. Toxicological analyses would be of value in correlating the concentration of substance metabolites in body fluids to the severity of anaemia. Finally, biochemical investigations such as iron, vitamin B12 and folate studies would be critical in distinguishing nutritional deficiencies due to intake and/or bioavailability. Investigating indirect bilirubin levels and haemoglobinuria would have added important information in characterizing haemolysis.

This study provides a detailed description of erythrocyte changes associated with anaemia in HIV-positive and HIV-negative injectable substance users. This information is valuable for the effectual clinical treatment and management of anaemia, more so in these groups who suffer from unresolved and recurrent episodes of anaemia despite of commencing treatment. The early and accurate diagnosis of dimorphic anaemia is important such that treatment may be effective amongst injection substance users. Thus, it is desirable that the clinico-haematological diagnosis of anaemia be accompanied by the critical evaluation of erythrocyte morphology on thin blood smears. Current practices in the diagnosis of anaemia rely majorly on the estimation of haemoglobin concentration which alone is inadequate to accurately identify dimorphic and other types of anaemia with adverse consequences on the choice of method for anaemia treatment and management. Therefore, regardless of the technological advancement in medical diagnostics, the accurate evaluation of thin blood films remains an essential tool in diagnosing an array of haematologic disorders and a reference for further tests and patient management.

## Conclusion and Recommendations

Infection with HIV is associated with increased risk of anaemia in injectable and non-injectable substance users. Majority of the anaemia was dimorphic suggestive of multiple aetiologies. Establishing the respective aetiologies is essential for the effective management of anaemia in illicit substance users. The accurate evaluation of thin blood films remains an essential tool in diagnosing an array of haematologic disorders and as a reference for further tests and patient management.

## Data statement

All additional files and supporting data are available from the Fig share database: ?10.6084/m9.figshare.7145870
